# Interplay between RNA interference and transposable elements in mammals

**DOI:** 10.3389/fimmu.2023.1212086

**Published:** 2023-07-05

**Authors:** Alexis Cornec, Enzo Z. Poirier

**Affiliations:** Stem Cell Immunity Team, Institut Curie, PSL Research University, INSERM U932, Paris, France

**Keywords:** RNA interference, mammals, transposable elements, Dicer (Dicer1), epigenetics, pattern recognition receptor (PRR), pathogen-associated molecular pattern (PAMP)

## Abstract

RNA interference (RNAi) plays pleiotropic roles in animal cells, from the post-transcriptional control of gene expression via the production of micro-RNAs, to the inhibition of RNA virus infection. We discuss here the role of RNAi in regulating the expression of self RNAs, and particularly transposable elements (TEs), which are genomic sequences capable of influencing gene expression and disrupting genome architecture. Dicer proteins act as the entry point of the RNAi pathway by detecting and degrading RNA of TE origin, ultimately leading to TE silencing. RNAi similarly targets cellular RNAs such as repeats transcribed from centrosomes. Dicer proteins are thus nucleic acid sensors that recognize self RNA in the form of double-stranded RNA, and trigger a silencing RNA interference response.

## Introduction

RNA interference (RNAi) is a ubiquitous mechanism of post-transcriptional regulation of gene expression (1). Present in plants, fungi and animals, it relies on the inhibition of messenger RNA translation via the production of micro-RNAs (miRNAs), a family of small RNAs. Irrespective of its function in regulating gene expression, RNAi also carries a role in antiviral immunity (2, 3). The machinery of RNAi is indeed equipped to recognize exogenous viral RNA and target it for cleavage, thereby thwarting infection. If exogenous virus infection represent an obvious threat, cell viability can also be jeopardized by the presence of transposable elements (TEs) in genomic DNA, which have the ability to disrupt gene expression regulation and genomic architecture (4, 5). To avoid such adverse effects, TEs are usually maintained transcriptionally silent via several mechanisms, including RNAi (6, 7). This review discusses the interplay between the machinery of RNAi and endogenous RNAs, including TEs. We focus here on mammals, without detailing the well-documented role of RNAi in controlling TEs present in invertebrates or plants [reviewed in (8)].

## The RNAi machinery as a pattern recognition receptor

**Figure 1:**
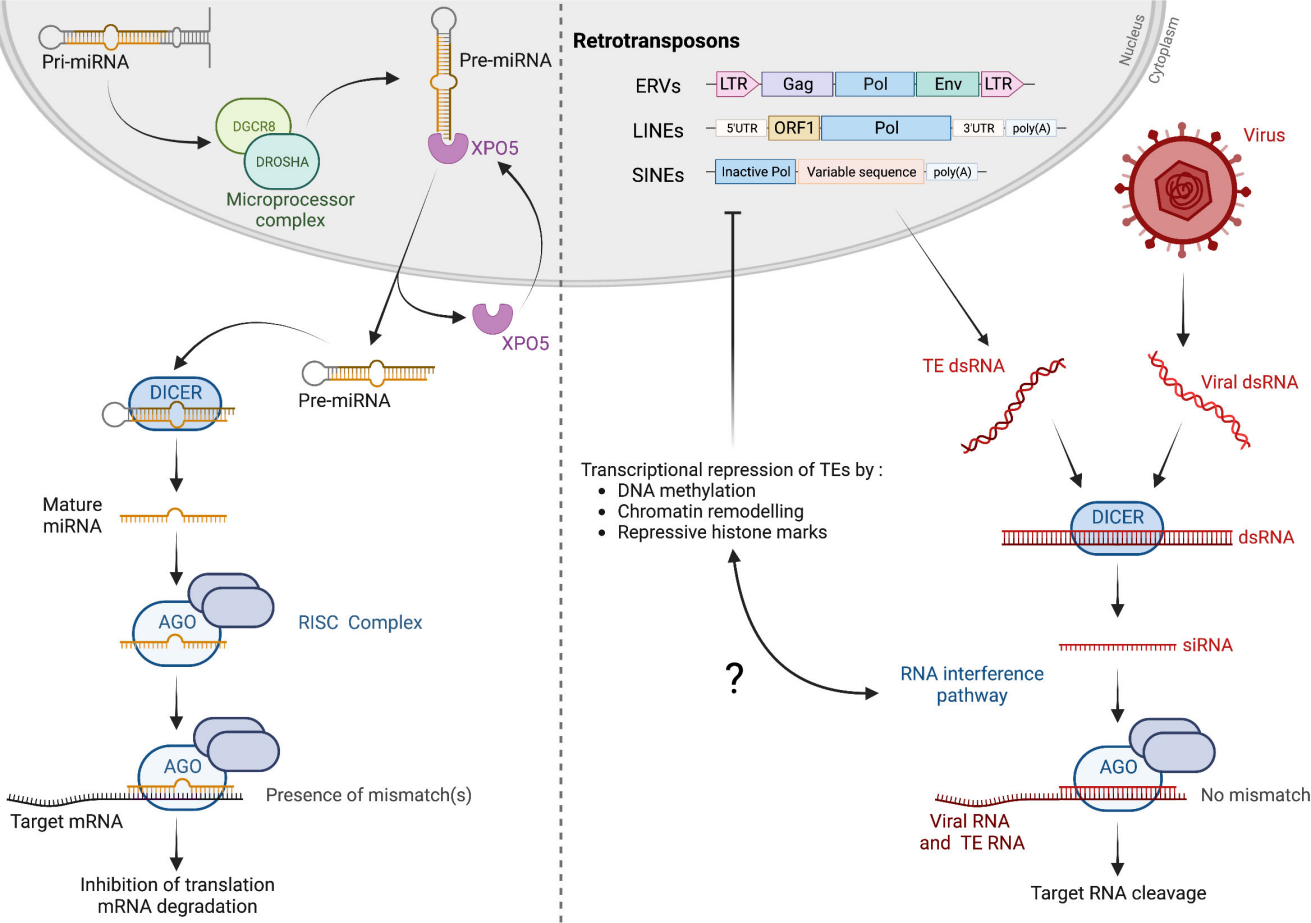
RNA interference in mammals. Left, post-transcriptional regulation of gene expression by RNAi. Pri-miRNAs, produced by genomic transcription, are processed into pre-miRNAs by the Microprocessor complex, composed of Drosha and Dgcr8. After shuffling to the cytoplasm, pre-miRNAs are cleaved by Dicer to generate mature miRNAs of 22 nucleotides in length on average. Loaded into RISC containing the endonuclease Ago, miRNAs guide the sequence-specific interaction with mRNAs to inhibit translation, resulting in mRNA degradation. Mismatch(s) between miRNA and mRNA sequences prevent degradation of mRNAs by Ago2. Right, expression of TEs, or RNA virus infection, lead to the cytosolic accumulation of dsRNA, which is processed by Dicer proteins, giving rise to siRNAs that guide cognate RNA degradation (no mismatch, Ago2 endonuclease is active). TEs are mainly composed of ERVs, LINEs and SINEs. ERVs may encode the ORFs Gag, Pol and Env, delimited by LTRs, while LINEs encode two ORFs. SINEs do not encode any functional ORFs and rely instead on LINE proteins for cycle completion.

Mammalian control of gene expression by the machinery of RNAi depends on the production of primary miRNAs (pri-miRNAs) by genomic transcription, which are cleaved in the nucleus by Drosha, generating precursor miRNAs (pre-miRNAs, **Figure 1**) (1). After being translocated to the cytoplasm, pre-miRNAs are processed by a protein of the Dicer family, giving rise to mature miRNAs. Incorporated into an RNA-induced silencing complex (RISC), miRNAs guide the interaction of Argonaute (Ago) with cognate mRNAs to inhibit translation (**Figure 1**). Embryonic lethality of Dicer knock-out mice highlights the physiological importance of this regulatory mechanism (9). The RNAi machinery is additionally involved in the protection against RNA viruses, playing a cornerstone antiviral role in multiple eukaryotes, including plants, worms and insects (3, 10–12). The defensive role of RNAi in mammals and humans has been debated, but recent data indicate that the pathway can be protective in certain cellular contexts. For example, antiviral RNAi protects mammalian stem cells from RNA viruses (13). Viral infection leads to the accumulation of double-stranded RNA (dsRNA) in the cytoplasm, originating from viral replication or from RNA secondary structures within viral genomes. Dicer cleaves dsRNA into small-interfering RNAs (siRNAs), which are small RNAs of approximately 21-23 nucleotides in length (**Figure 1**). As part of RISC, siRNAs guide the degradation of viral genomes by the endonuclease Ago. Note that, while miRNA-driven RNAi does not canonically function through mRNA cleavage, antiviral RNAi largely depends on the enzymatic activity of mammalian Ago2 (3). Mammals encode a single *Dicer* gene, which protein product, Dicer, generates miRNAs for transcription regulation. If several studies document human Dicer’s poor ability to target dsRNA for cleavage, the protein has been involved in dsRNA-driven antiviral RNAi in specific conditions (3, 14, 15). An additional isoform, termed antiviral Dicer (aviD), is produced by alternative splicing of mRNAs from the *Dicer* gene. aviD is expressed in stem cells within tissues and is specialized in targeting viral dsRNA as a starting point for antiviral RNAi (13). Another truncated isoform of Dicer, Dicer°, specific to rodents, is produced in oocytes and targets dsRNA (16).

An efficient antiviral RNAi response depends on the ability to specifically detect viral RNA, but not cellular RNA. This classical immunology conundrum of discrimination between “self” and “non-self” is achieved by targeting dsRNA, which represents a pathogen-associated molecular pattern (PAMP). In this context, Dicer, aviD and Dicer° display functional similarities with unrelated pattern recognition receptors (PRRs) that detect dsRNA, such as the interferon triggers RIG-I and MDA5 (17). In the absence of virus, formation of dsRNA structures within cellular RNAs is actively curtailed by Adar1, thereby preventing unwanted activation of immunity against “self” (18–21). Recognition of exogenous viral infection through dsRNA thus fits within the binary opposition “self” versus “non-self”. This is not the case when considering TEs, which are sequences –partly of retroviral origin– embedded in the genome. TEs could canonically be considered as “self” because they are encoded by the host, but nonetheless need to be identified and maintained transcriptionally silent.

## Transposable elements

Following the initial discovery of TEs by Barbara McClintock in 1950, decades of studies documented the quasi universal presence of TEs in organisms from the prokaryote and eukaryote kingdoms (22). TEs can be defined as genomic DNA sequences which, when intact, have the potential to express and re-integrate in the genome (23). While gene-encoding sequences account for 2% of total human genome, TEs constitute almost 50% of genomic DNA (23). Although regrouped under a single appellation, TEs are constituted of unrelated classes of elements, organized by their origin and transposition mechanism [**Figure 1** (24)]. Retrotransposons are TEs that perform a “copy and paste” cycle, which starts with TE transcription and RNA translocation in the cytoplasm. TE DNA is subsequently produced via reverse transcription, performed by a TE-encoded enzyme, before integration in genomic DNA. Retrotransposons make up for 90% of mammalian TEs, and are composed of two unrelated groups, identified by the presence or absence of long terminal repeats (LTR) [**Figure 1** (24)]. LTR elements originate from ancient events of germline infection by exogenous retroviruses; for this reason, they are coined endogenous retroviruses (ERVs) (25). Non-LTR elements are composed of long interspersed nuclear elements (LINEs) and short interspersed nuclear elements (SINEs), with LINEs being the most abundant TEs in mice (approximately 20% of the genome (26). In humans, the LINE-1 (L1) family was documented to be uniquely able to perform a complete cycle, including reverse transcription and integration (25). SINEs, which constitute about 10% of the mouse genome, do not encode an open reading frame, but rather rely on proteins encoded by LINEs. The human genome comports more than a million copies of a hominid-specific SINE termed Alu element, which can be transcribed and form dsRNA in the cytoplasm (27).

Amid the millions of TEs populating the mouse and human genomes, only a small fraction retains the ability to complete a full transposition cycle, which comprise the evolutionary young L1s in humans (4, 28). TE genomic neo-insertions can be a source of genetic innovation, by contributing to the organization of the tridimensional chromatin architecture, as well as by participating to the evolution of gene-regulatory networks (29, 30). Nonetheless, TEs represent a threat for genomic organization, as random TE insertions may occur within coding or regulatory sequences, influencing or disrupting gene expression (30). Indeed, TEs can act as enhancers or promoters for nearby genes, or can induce heterochromatin formation in the vicinity of insertions. In line with TEs’ deleterious effects, deficiencies in TE control have been or may be linked to cancer, neurodegenerative and developmental pathologies (4, 5, 31, 32). Inhibition of TE expression relies on multiple pathways, most of which act at the transcriptional level by inducing the formation of heterochromatin on TE loci. DNA methyltransferases, including Dnmt1, deposit methyl groups on cytosines within TE genomic DNA, providing binding sites for heterochromatin modifiers (6, 33–35). Histone methylation at specific positions, notably on lysine 9 of histone 3 (H3K9me3), represent another pathway of heterochromatin formation (36, 37). The use of a given mechanism of TE inhibition depends on the nature of the family/subfamily of TEs, as well as the cell type and the physiological/pathological context. Prevention of TE expression necessitates the specific recognition of TEs within the diversity of genomic sequences, akin to a distinction between “self” (coding and non-coding genes) and “non-self” (TEs). This partly depends on a family of proteins termed Krüppel-associated box zinc-finger proteins (KRAB-ZFPs) that evolved DNA-binding motifs recognizing TE genomic DNA in a sequence-specific manner (38–40). KRAB-ZFPs interact with cofactor KAP1/TRIM28 and guide the deposition of H3K9me3 on TE DNA by the histone methyltransferase SETDB1 (39, 41, 42). KRAB-driven recognition of TEs depends on the evolution of a TE-specific DNA binding motif, only possible across important evolutionary periods. Consequently, it is tempting to speculate that KRAB-ZFPs may not be able to target the entire spectrum of evolutionary young TEs, raising the question of the means by which such sequences are maintained transcriptionally silent.

## Interplay between the RNAi pathway and TEs

Soon after the initial discovery of RNAi by Andrew Fire and Craig Mello in *C. elegans*, data from the early 2000’s pointed towards a role of the pathway in controlling TEs in embryonic cells (43). Svoboda et al. used siRNAs and dsRNA injection in 1-cell embryos to knock-down Dicer, and demonstrated increased expression of Internal A Particles (IAP) and MERVL, two families of ERVs (44). In cultured human cells, siRNAs mapping to L1s could be detected, which production was linked to the activity of Dicer by a knock-down approach (45). Cumulative evidence similarly points towards a role of RNAi in controlling TE expression in embryonic stem cells (ESCs) (**Figure 1**). Knocking-out the *Dicer* gene in mouse ESCs and oocytes correlates with an increased expression of ERVs, LINEs and SINEs (16, 46–52). Additionally, TE expression is repressed by methylation of genomic DNA. Such heterochromatin mark is however erased during early development, upon a wave of demethylation occurring just after fertilization (53). Berrens et al. mimicked the wave of DNA demethylation in mouse ESCs by acutely depleting Dnmt1, leading to transcriptional activation of a limited set of TEs (52). In this context, RNAi participates to TE silencing in mouse ESCs, in compensation for the lack of methylation-driven transcription inhibition. Combining methylation loss and impaired RNAi translates into increased expression of a subset of evolutionary young ERVs such as IAPEz or MERVL (52). RNAi-driven control of TE expression is not solely present in undifferentiated cells such as ESCs. By studying age-related macular degeneration, a condition characterized by a progressive degradation of the retinal pigmented epithelium in aging patients, the Ambati group demonstrated that the phenotype is due to decreased Dicer levels, leading to upregulation of SINE RNAs (B1/B2 SINEs in mice and Alu elements in humans) (27). It is worth noting that the nature of TEs controlled by RNAi varies depending on the cell types and tissues considered. For example, Dicer ablation in cells of the retinal pigmented epithelium translates into increased TEs that seem largely restricted to SINEs, while LINEs and ERVs are upregulated in other cell types (27, 52). This could be related to the cell type-dependent organization of chromatin, linked to differentiation, as well as to other parameters.

In addition to Dicer-driven RNAi, data implicate piwi-interacting RNAs (piRNAs), a class of small RNAs, in the control of TEs (54, 55). The piRNA pathway is a well-documented anti-TE mechanism acting in the germline, through the generation of piRNAs transcribed from piRNA clusters (56). Recently, piRNAs, partly of TE origin, were detected in brain tissues of adult mice. Genetic ablation of the piRNA pathway translates into behavioral deficit, suggesting that it may be an additional small RNA-based mechanism that controls TE in somatic cells (54, 55). The functional importance of such mechanism, as well as the putative role of TE neo-integrations in piRNA clusters, is currently unknown.

## Molecular mechanism of TE control by RNAi

Dissection of the molecular mechanism behind RNAi control of TEs suggests that it follows a classical dsRNA-driven RNAi pathway (**Figure 1**). Knock-down and knock-out approaches targeting the Dicer transcripts/*Dicer* gene show that protein products of the *Dicer* gene are essential for the pathway. Upon loss of Dicer in the retinal pigmented epithelium, re-expression of TEs translates into accumulation of cytosolic dsRNA, detected by immunostaining using a specific antibody (27). If the TE sequences that form dsRNA await determination, the existence of TE dsRNA is also highlighted by a body of work showing that PRRs such as MDA5, which detect dsRNA and trigger an interferon response, can be activated upon TE expression in cancer cells (57–59). Formation of TE dsRNA is expected to arise from RNA secondary structures, from transcription of inverted repeats and from bidirectional transcription, when RNAs transcribed from both sense and antisense orientation hybridize (45, 50, 52). dsRNA from TE origin is presumably similar in structure to dsRNA generated upon exogenous infection by RNA viruses. The distinction between “self” and “non-self”, on which relies RNAi-driven control of TEs, thus appears akin to the specific detection of viral infection. Existence of siRNAs from TE origin, lost upon Dicer down-regulation or KO in mouse ESCs and oocytes, points towards a role of product(s) of the *Dicer* gene in cleaving TE dsRNA (16, 46–50, 52). siRNAs bearing TE sequences can be immunoprecipitated with Ago2, and Ago2 KO leads to increased TE expression, demonstrating that TE control depends on RISC activity (50, 52, 60). This is not universal, as Alu silencing in the retinal pigmented epithelium does not strictly depend on Ago2 (27). Note that, if canonical Dicer is the product of the *Dicer* gene deemed to be the main actor of dsRNA-driven control of TEs, other Dicer isoforms can participate in TE regulation, such as Dicer° in oocytes (16). Detection of dsRNA structures formed by TE RNA is regulated by Adar1, which prevents their binding by RLRs and the activation of an interferon response (20, 59). Whether Adar1 similarly dampens RNAi silencing of TEs is currently unknown. If data document RNAi control of TE expression through the detection of dsRNA, deviations from this mechanism exist. For example, small RNAs generated by Dicer processing of cellular transfer RNAs can participate to the silencing of certain ERV families (61). Specific miRNAs, such as miR-128 and let-7, regulate the L1 family of LINEs (62, 63). Irrespective of their detailed mechanism of control, inactivation of RNAi-driven TE control translates into increased TE expression, which can have dire functional consequences.

## Functional consequences of alleviating RNAi-driven control of TEs

Cytosolic dsRNA resulting from TE expression represents a canonical PAMP, or “non-self” signal, which, in differentiated cells, activates PRRs including MDA5 and the Toll-like receptor 3 (57, 58). Both PRRs’ stimulation results in the activation of innate immunity and production of interferons, potentially leading to autoimmunity. If pathways of interferon activation are functional in differentiated cells, they are severely compromised in stem cells, including ESCs, which could explain why TE re-expression does not translate into interferon-driven cytotoxicity in this context (3, 64). During age macular degeneration, expression of Alus in cells of the retinal pigmented epithelium leads to NLRP3 inflammasome activation and MyD88-dependent apoptosis, ultimately resulting in patient blindness (27, 65). Whether the downregulation of RNAi in pathological contexts could translate into TE re-expression, leading to inflammation and/or genomic instability, remains to be explored.

## Role of RNAi in processing other endogenous dsRNAs

RNAi-driven control of TEs, relying on dsRNA targeting, mirrors antiviral RNAi thwarting exogenous virus. Even if TEs are part of genomic DNA, one could, in this framework, consider TEs as “non-self”, controlled by the pathway of antiviral RNAi, even if most of the TEs are not from viral origin (ERVs, originating from ancient events of retrovirus integration, constitute around 8% of the human genome, to put in perspective with the 40% of virus-unrelated TEs). The binary distinction between “self” and “non-self” becomes even weaker when considering that a mechanism of RNAi-driven silencing of centromeric repeats has been documented. Centromeric repeats are indeed actively silenced via the RNAi-dependent deposition of heterochromatin marks (16, 66–70). Upon Dicer KO, expression of satellite repeats constitutive of centromeres correlates with major defects in mitosis and meiosis, resulting in impaired spermatogenesis. RNAi regulates expression of centromeric repeats via dicing of satellite dsRNA (69). dsRNA-driven RNAi is thus utilized in various settings, which include –but are not limited to– PAMPs such as TE dsRNA.

## Conclusions and perspectives

There is an established role for dsRNA-driven RNAi in the control of certain families of TEs, which bears strong similarities with the antiviral RNAi pathway acting against exogenous viral infections. In this pathway, Dicer proteins act as PRRs, recognizing PAMPs in the form of dsRNA of TE origin. If such mechanism shows efficiency in controlling TEs, the selective pressures behind its existence remain unclear. Indeed, more robust pathways such as DNA and histone methylation are at play in cells to sturdily prevent the expression of TEs. In that case, why use RNAi at all? Certain situations, such as the initial wave of DNA demethylation during embryonic development, call for compensatory mechanisms. In that case, RNAi fills the space left by the inactivation of the go-to mechanism of TE control. The existence of evolutionary young TEs, not yet targetable by sequence-specific KRAB-ZNFs, may be a second example of RNAi temporarily taking over, although this remains a speculation. Subfamilies of TEs such as IAPEz, ETn and MMERK10C, that are controlled by RNAi upon DNA methylation inhibition, are also the target of KRAB-ZNFs in mouse ESCs (40, 52). Whether RNAi synergizes with other pathways of TE regulation is currently unknown. Outstanding questions of the field include the thorough mapping of TE families controlled by RNAi, which are likely to be cell type and context-dependent, as well as the determination of TE sequences forming dsRNA. The regulation of TE expression participates in various physiological and pathological processes. Increased TE expression is involved, within normal physiology, in the establishment of an immune response against commensal microbiota (71). Cellular senescence, which correlates with aging, is associated with/can be driven by increased TE expression (72–74). Tumors express certain TEs at high levels, which bolsters anti-tumor immunity by triggering an inflammatory response via dsRNA detection, as well as by providing a source of neoantigens for adaptive immunity (75–79). Compounds able to awaken TE expression in tumors and boost immunity thus represent promising anti-cancer therapies (57–59, 80). Whether RNAi is involved in TE regulation in immunity, aging or cancer remains to be explored.

## Author contributions

EP and AC wrote the paper. AC designed the figure. All authors contributed to the article and approved the submitted version.
